# Supplementary material for: Vitamin A is necessary for acquisition, but not for expression or progression, of CNS autoimmunity
Source: bioRxiv. 2025 May 23:2025.05.18.654726. Preprint. [Version 1] doi: 10.1101/2025.05.18.654726 (PMC12148060; doi:10.1101/2025.05.18.654726)
Supplement: Supplement 1 [file media-1.pdf]

Suppl. Figure S1

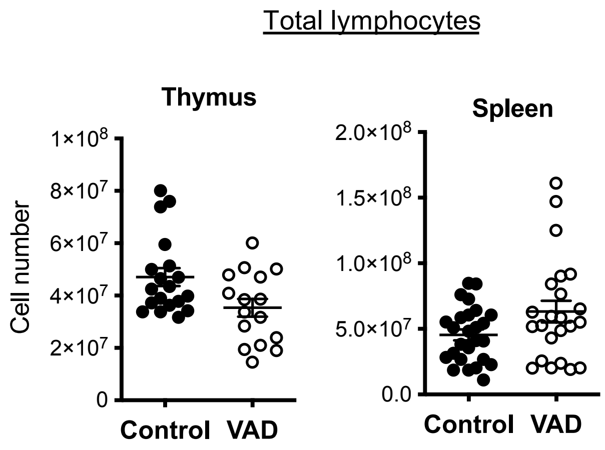

Suppl Figure 1. Total lymphocytes counts are unchanged in VAD mice.
Data are compiled from 6 to 8 experiments.

Suppl. Figure S2

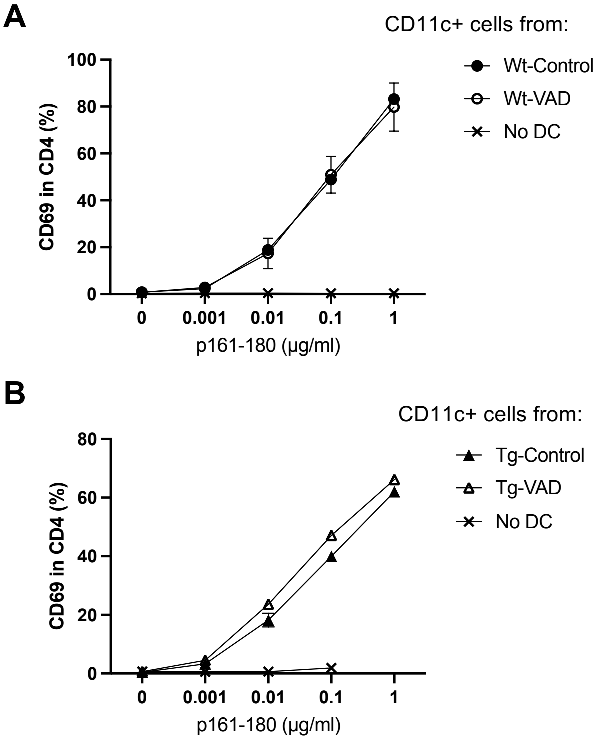

Suppl. Figure S2. CD11c+ dendritic cells from VAD mice are functionally intact in priming retina-specific T cells. Dendritic cells were isolated for the CD11c+ populations from spleen of VAD or control mice at 11 wk old or older age. Responder naïve CD4 T cells were purified from R161H Rag2$^{-/-}$ IRBP$^{-/-}$ mice. T cells and DC were co-cultured in the presence of varying concentrations of p161-180 for 20 h. (**A**) CD69 expression on the responder T cells cultured with WT CD11c+ cells. (**B**) CD69 expression on the responder T cells cultured with R161H CD11c+ cells. T cell only (No DC) wells served as negative controls. One representative experiment of 5 with 2-3 mice per group is shown.

Suppl. Figure S3

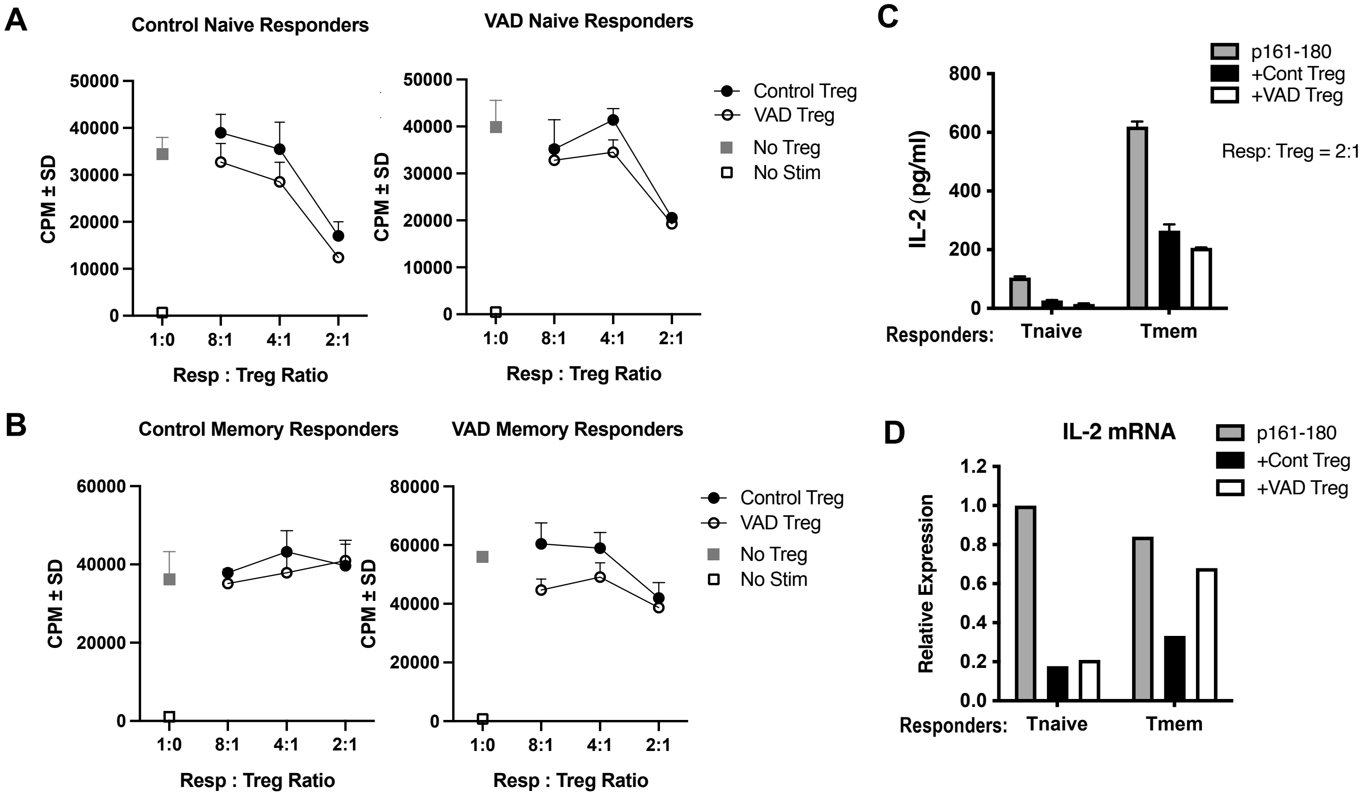

Suppl. Fig.S3. IRBP-specific Tregs from VAD and control mice have similar suppressive functions. Treg function from control and VAD R161H mice was evaluated in suppression assays with various numbers of Tregs in the co-culture. Naïve ($CD62L^{+}CD44^{lo}$) or memory ($CD62L^{-}CD44^{hi}$) responder (GFP-) or Treg (GFP+) cells were sorted from R161H-$Foxp3^{GFP}$ mice. Proliferative responses of (**A**) naïve responders from control or VAD mice or (**B**) memory responders from control or VAD mice, were measured in the absence or presence of Tregs with various ratios, cultured with antigen-presenting cells and IRBP161-180 peptide. (**C**) IL-2 concentration in culture supernatants after 18 h co-culture as analyzed by ELISA. (**D**) CD4+GFP- responder cells were sorted out 18 h after co-culture and analyzed for IL-2 mRNA. Relative expression to control Tn (without Treg) was calculated after normalization to respective GAPDH expression. (**C-D**) Responder to Tregs ratio was 2:1.

Suppl. Figure S4

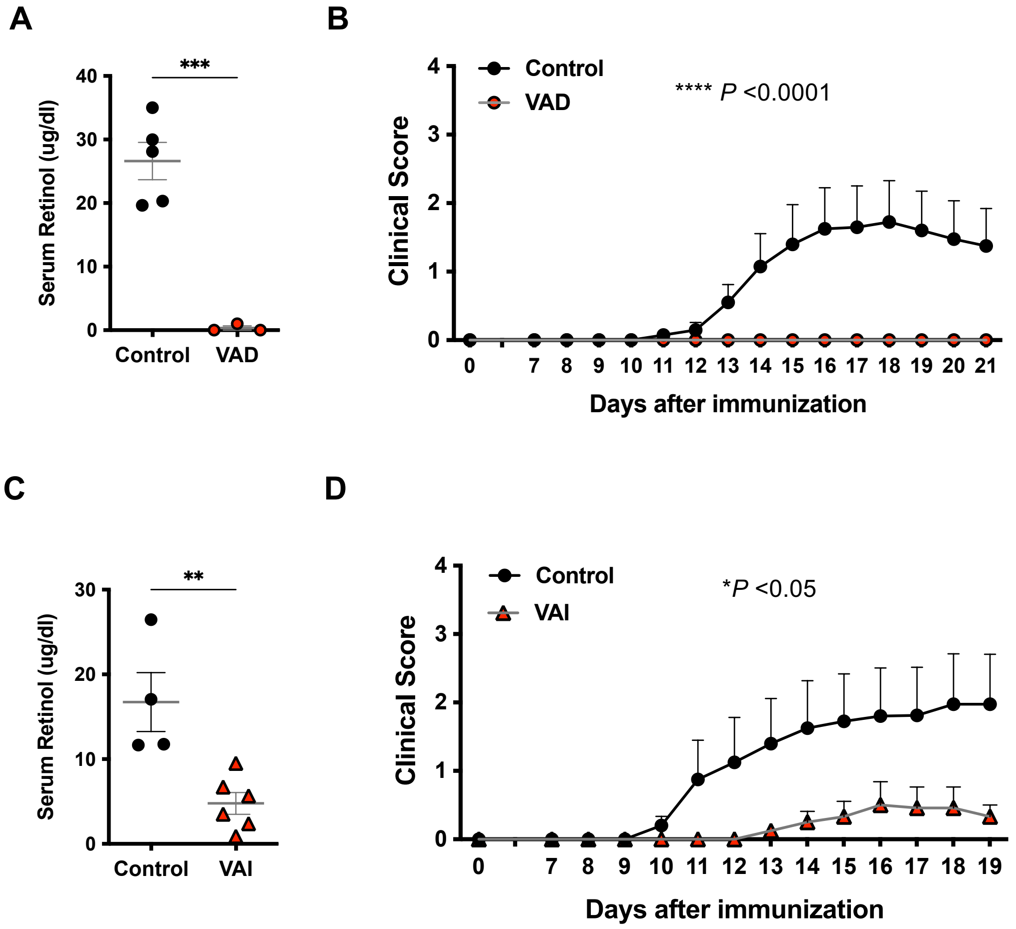

Suppl Figure S4. Induction of EAE is impaired in both complete and partial VAD mice. (**A,B**) B10.RIII mice were made VAD through dietary means and immunized at 9 wk of age with 300 µg MBP (whole protein) in 4 mg/ml CFA and two i.v injections of 0.2 µg PTX on day 0 and day 2. N=10 for Control and N=8 for VAD. Compiled data from two experiments. (**C,D**) Partial VAD (VAI) B10.RIII mice at 5 wk of age were immunized as described above. N=5 for Control, N=6 for VAD. One representative of two experiments. (**A, C**) Serum retinol were measured at the end of the experiments. ***P*<0.01, *** *P*<001 by unpaired t test.
